# Bioinformatics Identification and Functional Analysis of Key Genes of Nucleotide Metabolism in Oral Squamous Cell Carcinoma

**DOI:** 10.3290/j.ohpd.c_2470

**Published:** 2026-02-24

**Authors:** Feng Wei, Hong Fan

**Affiliations:** a Feng Wei Research Associate, Department of the First Clinical Division, Tianjin Stomatological Hospital, School of Medicine, Nankai University, No.73 Heiniucheng Road, Hexi District, Tianjin, 300041, China. Data analysis, drafting and revising the article, gave final approval of the version to be published, and was accountable for all aspects of the work.; b Hong Fan Research Associate, Department of the First Clinical Division, Tianjin Stomatological Hospital, School of Medicine, Nankai University, No.73 Heiniucheng Road, Hexi District, Tianjin, 300041, China. Data analysis, drafting and revising the article, gave final approval of the version to be published, and was accountable for all aspects of the work.

**Keywords:** bioinformatics analysis, gene set enrichment analysis, key genes, nucleotides, oral squamous cell carcinoma

## Abstract

**Purpose:**

Oral squamous cell carcinoma (OSCC) is a typical hypoxic and metabolically heterogeneous adult invasive oral malignant tumour. Nucleotide metabolism-related genes (NMRGs) have been identified as therapeutic targets for various cancers. This study aims to analyse the characteristics of NMRGs in OSCC and identify potential biomarkers.

**Materials and Methods:**

Based on the the Cancer Genome Atlas (TCGA) and GEO data, differentially expressed genes (DEGs) in OSCC were screened and intersected with NMRGs to obtain DE-NMRGs. Key genes were selected through the protein interaction (PPI) network combined with MCC and MCODE algorithms, and receiver operating characteristic (ROC) and survival curves were drawn. Genes with an area under the curve (AUC) > 0.7 and statistically significant differences were selected as hub genes. Further analysis of the immune infiltration characteristics, gene set enrichment analysis (GSEA) enrichment, potential drug effects of hub genes, and construction of the ceRNA network were conducted.

**Results:**

Three hub genes related to nucleotide metabolism (*ADA*, *NT5E*, and *TYMS*) were identified, showing good diagnostic performance (AUC > 0.7). Immune analysis showed that cytotoxic lymphocytes, B lineage, and monocytic lineage had increased infiltration in OSCC (P < 0.05). The ceRNA network showed that hsa-miR-30a-5p, hsa-miR-30b-5p interacted with *NT5E*, and hsa-miR-192-5p, hsa-miR-215-5p interacted with *TYMS*. Drug prediction suggested that denileukin difitox ontak, STREPTOZOCIN, and nitrogen mustard may be potential therapeutic drugs for OSCC.

**Conclusion:**

*ADA*, *NT5E* and *TYMS* can serve as potential diagnostic markers and therapeutic targets for OSCC. The study has reference value for early diagnosis and the development of individualised treatment strategies.

Oral squamous cell carcinoma (OSCC) is the most common type of oral epithelial tumour, with consistently high incidence and mortality rates worldwide.^48^ The occurrence of OSCC is closely related to poor dietary habits, smoking, alcohol consumption, poor oral hygiene, and chewing betel nuts.^5,15^ Additionally, chronic periodontitis and oral microecological imbalance can promote tumour development through inflammatory pathways, immune regulation, and changes in gene expression.^6,54,63^ Viral infections (such as HPV and EBV) are also considered important oncogenic factors for OSCC, participating in tumour development by interfering with host immune responses and regulating cell cycles.^9^ Immune cells such as oral epithelial sentinel cells (Langerhans cells) and γδT cells play a crucial role in the immune microenvironment of OSCC, and their dysfunction may lead to tumour immune escape, while natural defence proteins may regulate the immune microenvironment of precancerous lesions and tumours.^16,56^ Early diagnosis and disease monitoring of OSCC, as well as liquid biopsy and biomarker detection, provide new non-invasive strategies, including protein, RNA, and microbial-related markers, for the early diagnosis, prognosis assessment, and individualised treatment of OSCC.^24,44^ However, currently, surgical resection combined with radiotherapy and chemotherapy remains the commonly used treatment approach.^20^ Due to the complex anatomy, high rate of positive surgical margins, and early occult metastasis, the risk of postoperative recurrence is still high, with a five-year survival rate of only 50–60%.^42,52,53^ Therefore, exploring new molecular markers for early diagnosis, prognosis assessment, and precise treatment is crucial for improving the clinical management of OSCC.

Nucleotides, as fundamental units of genetic information, play pivotal roles in biological systems by functioning as building blocks of DNA/RNA, mediating cellular signalling, regulating enzyme activity, and maintaining metabolic homeostasis to ensure the normal functioning of the body. The malignant behaviours, including uncontrolled proliferation, chemoresistance, immune evasion, and metastatic potential in cancers such as OSCC, are largely sustained by enhanced nucleotide metabolism.^31^ The connections between nucleotide metabolism and OSCC progression have been established. For instance, the downregulation of Bax expression was shown to enhance chemosensitivity in OSCC-3. Zhang et al^62^ demonstrated that Drp1 suppression constitutes a key mechanism through which alantolactone induces apoptosis and represses proliferation in OSCC cells. Additionally, Zou et al^65^ reported that overexpression of long non-coding RNA lncC00472 suppresses OSCC malignant progression. Current research has primarily focused on developing nucleotide metabolism enzyme inhibitors and applying metabolomics in OSCC studies.^18,55^ There is still insufficient research on how these genes affect the occurrence of OSCC, as well as their signalling pathways and immune infiltration in tumour biology. Based on the existing literature suggesting that NMRGs play a crucial role in the occurrence and development of various cancers, we hypothesise that NMRGs also have important biological functions in OSCC and may have potential diagnostic value.

This study aims to identify the key NMRGs in OSCC based on systematically integrated data from the TCGA and GEO databases, analyse their immunological correlations and potential drug targets, and construct a ceRNA network, providing new molecular targets and research directions for the diagnosis and personalised treatment of OSCC.

## MATERIALS AND METHODS

### Data Acquisition

Gene expression profiles and corresponding clinical data (including age, gender, tumour grade, and TNM stage) of OSCC samples were retrieved from the Cancer Genome Atlas (TCGA) database. The TCGA-OSCC cohort, comprising 32 normal samples and 341 tumour samples, served as the training set. Samples with survival times exceeding 30 days were retained for survival analysis. Two independent OSCC-related data sets (collectively referred to as GEO-OSCC) were downloaded from the GEO database (https://www.ncbi.nlm.nih.gov/geo/) as the validation set, including: GSE25099 (22 normal samples, 57 OSCC samples) and GSE30784 (45 normal samples, 167 OSCC samples). The TCGA expression matrix was processed for missing value imputation, average of duplicate genes, filtering of low-expression genes (average count ≤ 1), and was standardised by using the TMM method of edgeR. Subsequently, the dispersion was estimated using a negative binomial distribution. The precise tests were conducted to screen for DEGs. All downstream analyses were based on the matrix standardised by TMM. The GEO data was already normalised by the authors by using quantile normalisation and log2 transformation, and could be directly used without further standardisation. NMRGs (Table A1) were obtained from the REACTOME_METABOLISM_OF_NUCLEOTIDES pathway in the Molecular Signatures Database (MSigDB).

### Identification of DE-NMRGs and Functional Enrichment Analysis

Differentially expressed genes (DEGs) between normal and tumour samples in TCGA-OSCC were analysed using the ‘edgeR’ package with thresholds of FDR < 0.05 and |logFC| > 1. The intersection of DEGs and NMRGs yielded differentially expressed NMRGs (DE-NMRGs). Subsequent Kyoto Encyclopedia of Genes and Genomes (KEGG) and Gene Ontology (GO) enrichment analyses were undertaken using the ‘ClusterProfiler’ package to elucidate potential functional pathways, with results visualised via the ‘enrichplot’ R package.

### Identification of Key DE-NMRGs

Protein-protein interaction (PPI) networks of DE-NMRGs were constructed using the STRING database with an interaction score threshold > 0.4. The CytoHubba plugin in Cytoscape software (version 3.10.0) was employed to identify hub nodes, with the top 10 key genes selected using the MCC method. The MCODE algorithm was applied to detect significant functional modules within the PPI network. Key genes were determined by intersecting results from the two algorithms. Expression patterns of key genes in the TCGA-OSCC cohort were visualised using boxplots generated by the ‘ggpubr’ R package. Pearson correlation coefficients between key genes were calculated and visualised as chord diagrams using the ‘circlise’ R package.

### Diagnostic Evaluation of Key Genes and Hub Gene Identification

Receiver operating characteristic (ROC) curves were generated, and area under the curve (AUC) values were calculated using the ‘pROC’ package to assess the diagnostic performance of key genes in TCGA-OSCC, which was validated in GEO-OSCC. The genes with AUC values greater than 0.7 in multiple data sets and showing significant differences between the tumour group and the control group were selected as the final hub genes for subsequent analysis.

To further explore the prognostic value of the genes, the ‘survminer’ R package was used to divide OSCC patients into high-expression and low-expression groups based on the median expression levels of the key genes, and survival analysis was conducted. Additionally, in the TCGA-OSCC data set, clinical factors such as age, gender, TNM stage, and tumour stage, as well as 10 hub genes, were included for univariate and multivariate Cox regression analyses to investigate the independent prognostic value of these genes.

### Immune Infiltration Analysis

The MCP counter algorithm implemented in the ‘IOBR’ R package was employed to assess differences in TME composition between normal and tumour samples in the TCGA-OSCC data set. The inter-group differences were compared using the Wilcoxon rank sum test. The correlation between hub genes and immune cells was calculated using the ‘Hmisc’ package (Pearson correlation analysis), and the results were visualised using ‘ggcorrplot’. The significance level was set at P < 0.05.

### Gene Set Enrichment Analysis (GSEA) of Hub Genes

Differential expression analysis was conducted on groups stratified by survival analysis using the ‘limma’ package. Subsequently, KEGG pathway enrichment analysis of DEGs was carried out with the ‘ClusterProfiler’ package. The top 10 significantly enriched pathways (q value < 0.05, adjusted P value < 0.05) ranked by normalised enrichment score (NES) were selected for visualisation.

### Correlation Analysis Between Hub Genes and OSCC-related Genes

The top 100 OSCC-related genes with the highest relevance scores were retrieved from GeneCards and were intersected with DEGs from TCGA-OSCC normal and tumour samples to identify OSCC-related genes. The ‘Hmisc’ package was used to analyse the correlations between these genes and the hub genes (through Pearson correlation analysis). The results were visualised using the ‘corrplot’ and ‘ggplot2’ packages, with the significance level set at P < 0.05.

### Construction of the ceRNA Regulatory Network

To identify the key lncRNAs, we conducted further analysis on them in the TCGA cohort. Firstly, we performed differential analysis of lncRNA expression data in the TCGA using the ‘edgeR’ package, with the data preprocessing and threshold setting being consistent with the analysis of mRNA. Subsequently, we took the intersection of the differentially expressed lncRNAs identified and those in the ceRNA network to obtain the common lncRNAs for subsequent analysis. Finally, we used the ‘survival’ R package to conduct univariate Cox proportional hazards regression to identify lncRNAs significantly associated with overall patient survival (P < 0.05).

### Drug Prediction

The drug sensitivity data were obtained from the CellMiner database (https://discover.nci.nih.gov/cellminer/home.do), and the correlation between hub genes and drug responses (half inhibitory concentration, IC50) was calculated (using Pearson correlation analysis). Subsequently, the top 12 drugs with the strongest correlation to the hub genes were selected and visualised using ‘ggplot2’. The significance level was set at P < 0.05.

## RESULTS

### Identification of DE-NMRGs and Enrichment Analyses

**Fig 1a to c:**
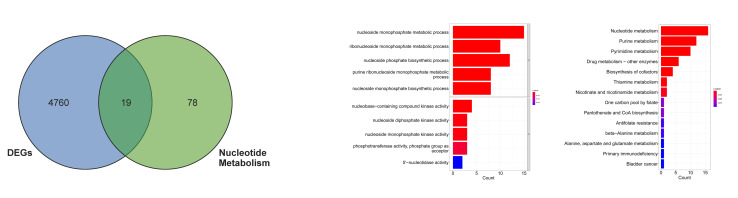

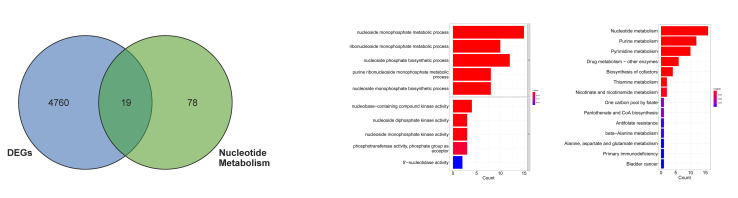
Identification and KEGG and GO analyses of DE-NMRGs. (a) Venn diagrams of DEGs and NMRGs in normal samples and cancer samples in TCGA-OSCC. (b) GO enrichment analysis of DE-NMRGs. BP: biological process; MF: molecular function. (c) KEGG enrichment analysis of DE-NMRGs.

The intersection analysis between DEGs in TCGA-OSCC normal and tumour samples with 97 NMRGs yielded 19 DE-NMRGs (Table A2, Fig 1a). GO enrichment analysis revealed that DE-NMRGs were predominantly enriched in biological processes, including nucleoside monophosphate metabolic process, ribonucleoside monophosphate metabolic process, and nucleoside phosphate biosynthetic process, as well as molecular functions such as nucleobase-containing compound kinase activity, nucleoside diphosphate kinase activity, and nucleoside monophosphate kinase activity (Fig 1b). KEGG pathway analysis demonstrated significant enrichment of DE-NMRGs in nucleotide metabolism, purine metabolism, and pyrimidine metabolism pathways (Fig 1c).

### Identification Key DE-NMRGs

**Fig 2a to f:**
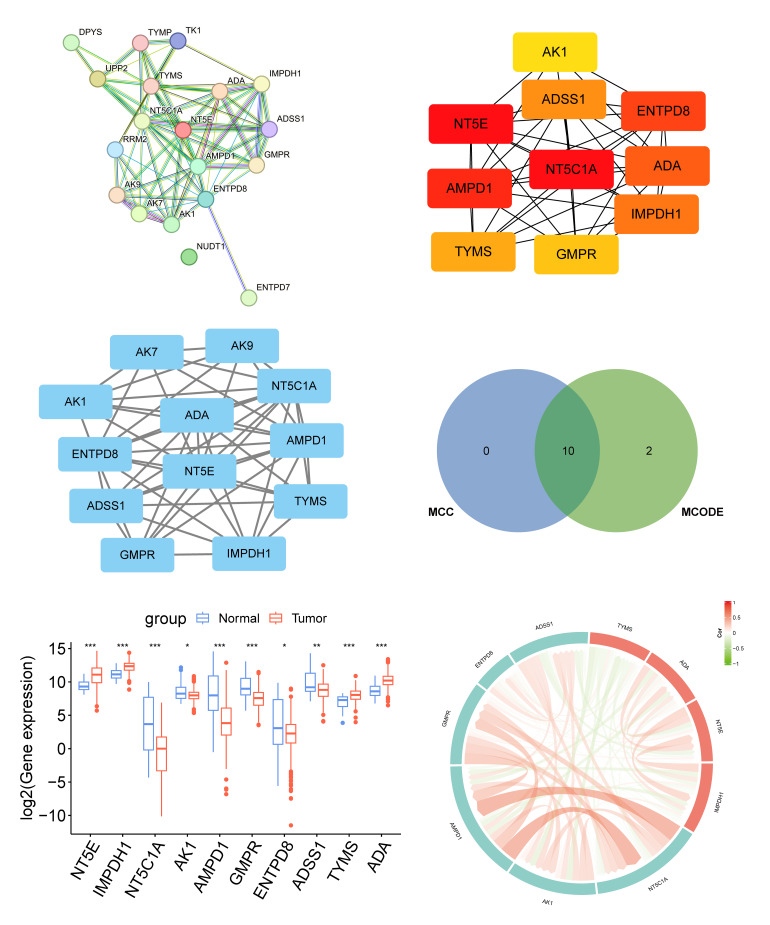
Identification of key DE-NMRGs and their expression in normal and cancer samples in TCGA-OSCC. (a) PPI network of DE-NMRGs in TCGA-OSCC. (b) Hub genes identified by the MCC algorithm. (c) Hub genes identified by the MCODE algorithm. (d) Venn diagram of hub genes identified by MCC and MCODE algorithms. (e) Expression of key genes in TCGA-OSCC normal and cancer samples. (f) Correlation map between key genes. *signifies P < 0.05; **signifies P < 0.01; ***signifies P < 0.001; ns signifies not significant.

PPI network revealed complex interaction patterns among DE-NMRGs (Fig 2a). Intersection analysis between the top 10 genes identified by the MCC algorithm (Fig 2b) and 12 significant genes detected by the MCODE algorithm (Fig 2c) yielded 10 key genes (Fig 2d). Among these, *NT5E*, *IMPDH1*, *TYMS*, and *ADA* showed significantly elevated expression in tumour tissues, while the remaining six genes (*NT5C1A*, *AK1*, *AMPD1*, *GMPR*, *ENTPD8*, and *ADSS1*) exhibited downregulation (Fig 2e). Correlation analysis demonstrated particularly strong co-expression between *AMPD1* and *NT5C1A* (Cor > 0.5) (Fig 2f).

### Diagnostic Performance and Survival Analysis of Key Genes

**Fig 3a and b:**
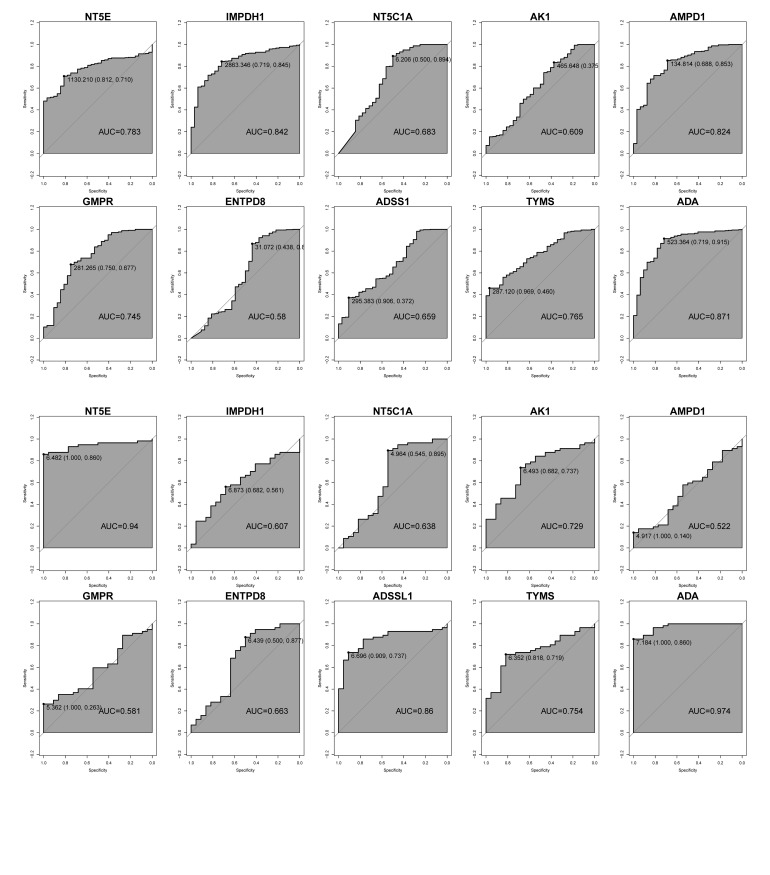
Diagnostic capabilities of key genes in TCGA-OSCC and GEO-OSCC (GSE25099). (a) ROC curve of key genes in TCGA-OSCC queue. (b) ROC curve of key genes in the GEO-OSCC (GSE25099) queue.

To evaluate the diagnostic capabilities of the key genes, we plotted the ROC curves for 10 key genes in TCGA-OSCC and GEO-OSCC (GSE25099 and GSE30784). The AUC values of the key genes *IMPDH1*, *AMPD1*, and *ADA* in TCGA-OSCC were higher than 0.8. The AUC values of *NT5E*, *GMPR*, and *TYMS* were higher than 0.7, and the AUC value of the gene *ENTPD8* was the lowest (0.58) (Fig 3a). In GSE25099, the AUC values of the key genes* NT5E*, *ADSS1* (*ADSSL1*), and *ADA* were higher than 0.8. The AUC values of *AK1* and *TYMS* were higher than 0.7, and the AUC value of the gene *AMPD1* was the lowest (0.522) (Fig 3b). In GSE30784, the AUC values of the key genes *IMPDH1*, *AMPD1*, and *ADA* were higher than 0.7; the AUC value of the gene *NT5C1A* was the lowest (0.559) (Appendix Figure 1). Among them, the AUC values of *NT5E*, *ADA*, *TYMS*, and *AMPD1* were all higher than 0.7 in multiple cohorts, suggesting that these genes had good diagnostic performance. Further differential expression analysis revealed that *NT5E*, *ADSS1*, *ADA*, and *TYMS* were differentially expressed between tumour and normal samples in GSE25099 and GSE30784 Appendix Figure 2a and 2b). Based on the AUC performance and differential expression results, *ADA*, *NT5E*, and *TYMS*, which had high AUC values and were differentially expressed in all three data sets, were determined as hub genes with high comprehensive diagnostic value.

**Fig 4:**
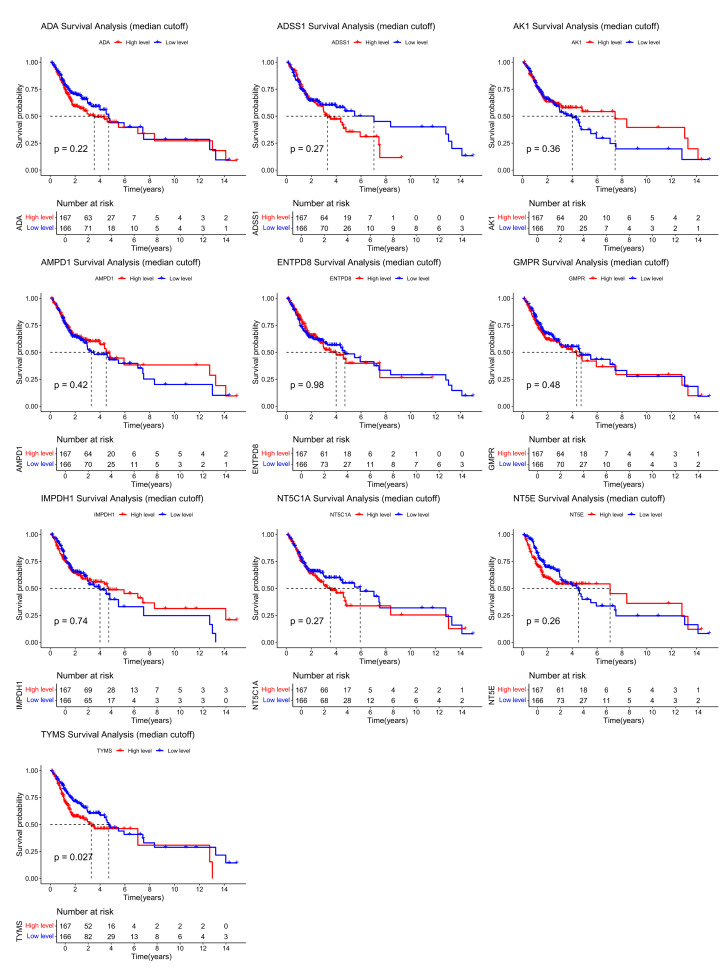
Survival analysis of groups based on the median expression values of key genes in TCGA-OSCC.

In addition, to explore the relationship between the key genes and patient prognosis, we divided the tumour samples into high- and low-expression groups based on the median expression levels of each gene in TCGA-OSCC for survival analysis (Fig 4). The results showed that there was a statistically significan toverall survival difference in the *TYMS* high-expression group (P < 0.05), while the survival curves of other genes did not reach a statistically significant level, suggesting that *TYMS* may be related to patient prognosis. Further univariate and multivariate Cox regression analyses showed that only *ADA* had independent prognostic value (P < 0.05) among these genes (Appendix Figure 2c and 2d). Therefore, based on these analyses, we identified *ADA*, *NT5E*, and *TYMS* as hub genes with high diagnostic value, but their prognostic value is not yet clear.

### Immune Infiltration Analysis

**Fig 5a and b:**
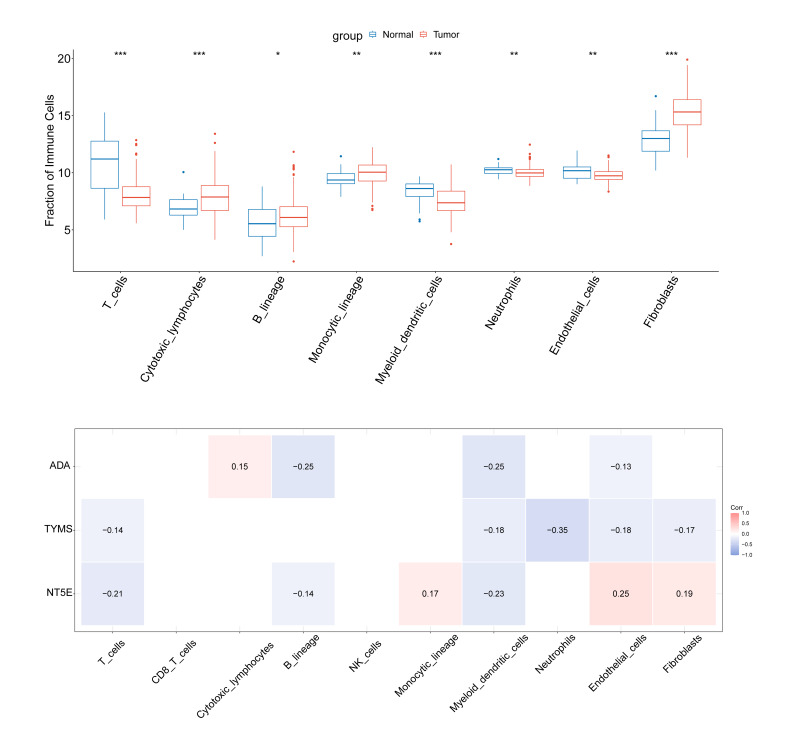
Immune cell invasion analysis and association analysis of hub genes with immune cells in normal and cancer samples in TCGA-OSCC. (a) Immune cell infiltration analysis of normal and cancer samples in TCGA-OSCC. (b) Correlation between hub genes and immune cells. *signifies P < 0.05; **signifies P < 0.01; ***signifies P < 0.001; ns signifies not significant.

To evaluate differences in TME in normal and malignant tissues, immune infiltration analysis was conducted on the TCGA-OSCC cohort. Normal samples exhibited elevated infiltration of T cells, myeloid dendritic cells (mDCs), neutrophils, and endothelial cells (ECs), whereas OSCC samples showed higher infiltration of cytotoxic lymphocytes, B lineage cells, monocytic lineage cells, and fibroblasts (Fibs) (Fig 5a). The correlation analysis of hub genes and immune cells revealed that *ADA* was negatively linked with B lineage and mDCs (cor = –0.25) and positively linked with cytotoxic lymphocytes (cor = 0.15). *NT5E* exhibited a positive linkage with ECs (cor = 0.25) but an inverse linkage with mDCs (cor = –0.23). *TYMS* was inversely linked with neutrophils (cor = –0.35) (Fig 5b)

### GSEA of Hub Genes

**Fig 6a to f:**
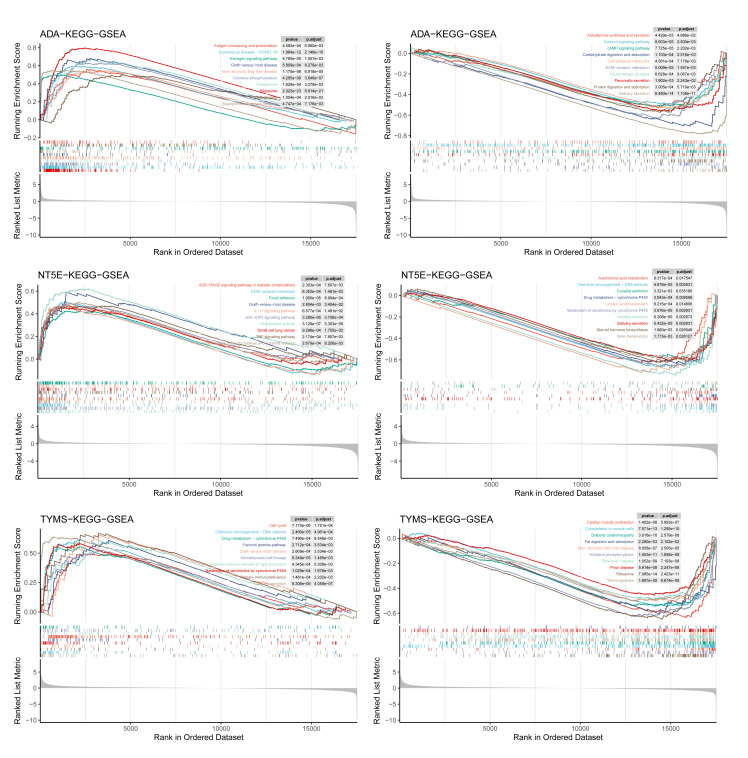
GSEA of DEGs of hub gene based on survival analysis. (a) GSEA results for upregulated DEGs determined by *ADA*. (b) GSEA results for downregulated DEGs determined by *ADA*. (c) GSEA results for upregulated DEGs determined by *NT5E*. (d) GSEA results for downregulated DEGs determined by *NT5E*. (e) GSEA results for upregulated DEGs determined by *TYMS*. (f) GSEA results for downregulated DEGs determined by *TYMS*.

Based on groups stratified in survival analysis on hub genes, we screened out the DEGs of the two groups. The groups based on *ADA* demonstrated that upregulated DEGs were enriched in pathways such as antigen processing/presentation, and oestrogen signalling pathway (Fig 6a). Downregulated DEGs were gathered in ECM–ECM-receptor interaction and cAMP signalling pathway (Fig 6b). The groups based on *NT5E* revealed that upregulated DEGs were enriched in the Toll-like receptor signalling pathway and IL-17 signalling pathway (Fig 6c), while downregulated DEGs were in chemical carcinogenesis-DNA adducts and arachidonic acid metabolism (Fig 6d). The groups based on *TYMS* showed that upregulated DEGs were concentrated in the cell cycle and Fanconi anaemia pathway (Fig 6e) and downregulated DEGs were in oxidative phosphorylation (Fig 6f).

### Correlation Analysis Between Hub Genes and OSCC-Related Genes

**Fig 7:**
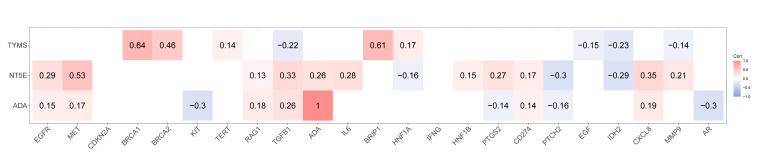
Correlation analysis of hub gene and OSCC-related gene.

Intersection analysis of DEGs in normal and cancer samples in TCGA-OSCC and the top 100 OSCC-related genes yielded 23 genes (Table A3). Correlation analysis revealed statistically significant positive associations (|cor|>0.2) between hub gene *ADA* and disease-related gene *TGFB1*, which was also found between *NT5E* and *MET*, *TGFB1* and *CXCL8*, *TYMS* and *BRCA1*, as well as between *BRCA2* and *BRIP1*. Moreover, *ADA* was inversely linked with KIT and AR (|cor|<-0.2). *NT5E* was negatively linked with *PTCH2* and *IDH2* (|cor|<-0.2). *TYMS* had inverse linkages with *TGFB1* and IDH2 (|cor|<-0.2) (Fig 7).

### Construction of the ceRNA Regulatory Network of Hub Genes

**Fig 8:**
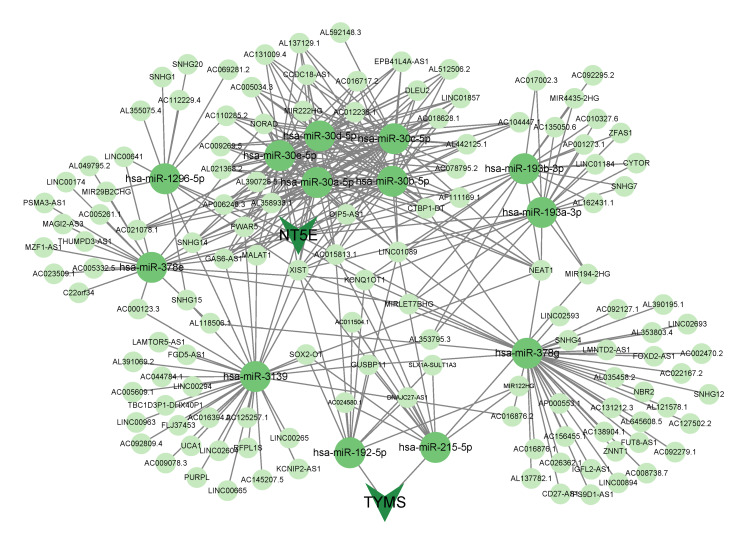
ceRNA network of hub genes.

A total of 42 miRNAs that interacted with three hub genes were detected in the mirDIP database, with 13 miRNAs being detected in the ENCORI database. No miRNAs regulating *ADA* genes were detected. *NT5E* interacted with hsa-miR-30a-5p, hsa-miR-30b-5p, hsa-miR-30c-5p, hsa-miR-30d-5p, hsa-miR-30e-5p, hsa-miR-193a-3p, hsa-miR-193b-3p, hsa-miR-378e, hsa-miR-378 g, hsa-miR-1296-5p, hsa-miR-3139. Both hsa-miR-192-5p and hsa-miR-215-5p interacted with *TYMS*. A total of 130 lncRNAs were predicted to interact with these 13 miRNAs using the ENCORI database (Table A4). Finally, a ceRNA network was constructed using Cytoscape (Fig 8).

To identify the key lncRNAs, we conducted further analysis on them in the TCGA cohort. By performing differential expression analysis on the lncRNAs in the TCGA data set, we obtained 2845 differentially expressed lncRNAs. Subsequently, we took the intersection of these 2845 lncRNAs with the 130 lncRNAs predicted by the ENCORI database, resulting in 22 intersecting lncRNAs. Univariate Cox regression analysis revealed that two of these 22 lncRNAs (FOXD2.AS1 and AC156455.1) were significantly associated with patients’ overall survival (P < 0.05). Kaplan–Meier survival analysis demonstrated astatistically significant difference in survival between patients with high- and low-expression levels of FOXD2.AS1 and AC156455.1 (P < 0.05) (Appendix Figure 3).

### Drug Prediction

**Fig 9:**
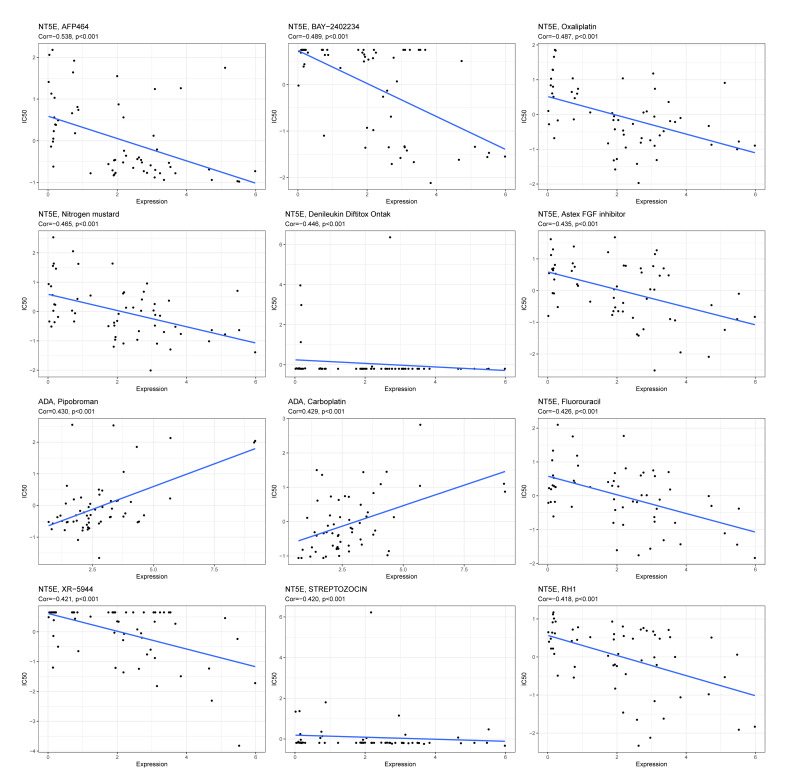
Correlation between hub genes and drugs.

**Fig 10:**
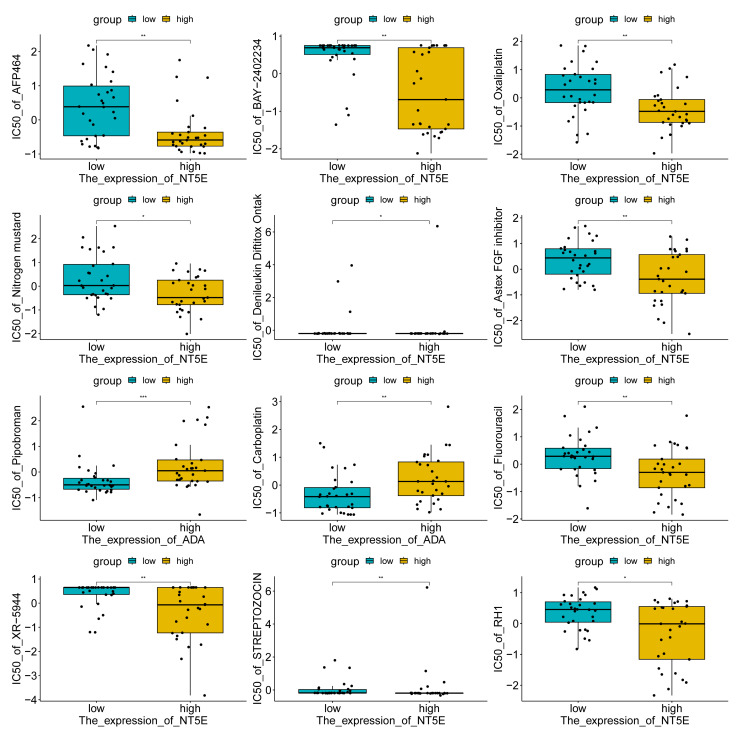
Sensitivity map of hub genes and drugs. *signifies P < 0.05; **signifies P < 0.01; ***signifies P < 0.001; ns signifies not significant.

The drug sensitivity prediction results based on the CellMiner database showed that the IC50 values of drugs AFP464, BAY-2402234, oxaliplatin, nitrogen mustard, denileukin diftitox (Ontak), Astex FGF inhibitor, fluorouracil, XR-5944, STREPTOZOCIN, and RH1 decreased as the *NT5E* expression level increased (P < 0.001), suggesting that samples with high *NT5E* expression may have higher sensitivity to these drugs. Conversely, the IC50 values of drugs pipobroman and carboplatin increased as the *ADA* expression level increased (P < 0.001), indicating that samples with low *ADA* expression may be more sensitive to these drugs (Figs 9 and 10). These results were based on the computer simulation correlation analysis of the CellMiner platform and could only provide predictive clues for potential drug responses. Further experiments or clinical validations are required.

## DISCUSSION

OSCC, the most prevalent oral cancer, is characterised by a dismal prognosis and high mortality rates.^55^ We herein systematically identified NMRGs in OSCC and validated hub genes with superior diagnostic potential. Comprehensive immune infiltration analysis revealed significant correlations between hub genes and specific immune cells. *ADA* was positively linked with B lineage and mDCs. *NT5E* had shown a positive relationship with ECs (cor >0.2). *TYMS* was inversely linked with neutrophils (cor <-0.2). The same inverse linkages were detected between *NT5E* and T cells and mDCs (cor <-0.2). We also predicted potential drugs that target hub genes, demonstrating that denileukin diftitox ontak, STREPTOZOCIN, and nitrogen mustard were promising drugs for OSCC treatment.

In our study, three hub genes related to NMRGs, namely *ADA*, *NT5E*, and *TYMS*, were identified. These genes showed high AUC values in multiple independent data sets, suggesting that they might serve as potential diagnostic markers for OSCC. Adenosine deaminase (ADA) catalyses the conversion of adenosine to inosine in the purine degradation pathway^38^ and is significantly elevated in HNSCC tissues. Its high expression is associated with poor survival outcomes.^12,22,64^ In this study, the high expression of *ADA* group upregulated genes were enriched in antigen processing and presentation and the oestrogen signalling pathway, while downregulated genes were enriched in ECM-receptor interaction and cAMP signalling pathway, suggesting that *ADA* might participate in the progression of OSCC by regulating tumour antigen presentation, cell proliferation, and microenvironment remodelling,^2,34,59^ which is consistent with previous study showing that *ADA* is involved in immune regulatory signals.^39^ Although *ADA* shows potential diagnostic value, this study did not verify its prognostic effect, and the clinical prognostic significance of this gene still needs further research to confirm. 5’-Nucleotidase Ecto (*NT5E*, CD73) is a membrane protein that can catalyse the conversion of extracellular nucleotides to membrane-permeable nucleotides^47^ and plays a key role in tumour growth and metastasis.^46^ Previous studies have found that high expression of *NT5E* in OSCC and HNSC is associated with poorer survival outcomes.^7,45^ The GSEA analysis in this study showed that upregulated genes in the high expression of the *NT5E* group were enriched in Toll-like receptors and IL-17 signalling pathways, suggesting that *NT5E* might affect the tumour microenvironment and immune escape through immune regulation and inflammatory mediator metabolism.^33,50,51^ Consistent with previous research,^43^ this study further suggests that *NT5E* has potential diagnostic value in tumours, but its role in the prognosis of OSCC still needs further verification. Thymidylate synthetase (TYMS) is a rate-limiting enzyme required for DNA synthesis and is a key target for various chemotherapy drugs (such as 5-FU, methotrexate).^17,28,36^ High expression of *TYMS* is associated with advanced progression of OSCC and 5-FU resistance,^1^ and its 28 bp tandem repeat polymorphism may affect the genetic susceptibility of head and neck cancer.^10^ In this study, upregulated genes in the high expression of *TYMS* group were enriched in the cell cycle and Fanconi anaemia pathway, while downregulated genes were enriched in oxidative phosphorylation, suggesting that it may promote DNA synthesis, regulate cell proliferation, and support the rapid growth of OSCC, which is consistent with the previously discovered mechanism of *TYMS* mediating tumour proliferation and chemotherapy resistance.^13,30,40^ Although identified hub genes showed good diagnostic efficacy, this study did not verify their prognostic value. In summary, *ADA*, *NT5E*, and *TYMS* all demonstrated high diagnostic efficacy in multiple independent cohorts. GSEA analysis suggested that they might participate in the occurrence and development of OSCC by regulating immune, inflammatory, and cell proliferation-related pathways. Notably, this study did not verify the prognostic role of these genes, and their clinical significance still needs to be further studied and confirmed.

In this study, we also analysed the infiltration of immune cells in normal tissues and cancer tissues. Cytotoxic T lymphocytes (CTL), B cell lineages, monocytic lineages, and fibroblasts had a higher infiltration degree in OSCC patients. This finding is consistent with existing studies. For example, John et al^19^ reported that the number of CD8+ T cells was significantly increased in OSCC and oral developmental disorders patients, further confirming the phenomenon of high infiltration of CTL in OSCC. However, immune suppressive factors in the tumour microenvironment (TME) can lead to CTL dysfunction and exhaustion, thereby promoting the formation of adaptive immune tolerance.^11^ In this context, the overall accumulation of tumour-infiltrating lymphocytes (TILs) was still confirmed to be related to better radiotherapy and chemotherapy responses and prognosis in OSCC patients,^27^ highlighting the significance of reversing immune suppression and activating the function of TILs. B cells, as the core members of humoral immunity, play a crucial role in tumour immunity.^4^ Kong et al^26^ found that *TP53* mutations may lead to a decrease in the infiltration degree of B cell lineages and other immune cells, thereby exerting a negative impact on the prognosis of HSNCC. Additionally, in OSCC, the increase in *TCL1A* expression level can promote the upregulation of complement receptor 2 on B cell surfaces, promoting the phagocytosis and antigen presentation ability of dendritic cells,^58^ suggesting that B cells play an active role in anti-tumour immunity. Moreover, the fusion of monocyte lineages/macrophages with tumour cells may promote tumour metastasis.^25^ Fibroblasts, especially cancer-associated fibroblasts (CAFs), are highly expressed with tumour-promoting cytokines and muscle fibroblast markers under the induction of *ANGPTL323*. The Mesen_CAF subgroup is significantly enriched in pathways such as TGF-β, EMT, angiogenesis, and PI3K-AKT-mTOR, exhibiting an important role in regulating the structure of the tumour microenvironment and promoting the malignant progression of OSCC-49. In addition to the characteristics of immune cell infiltration, this study found that NMRG-related hub genes (*ADA*, *NT5E*, *TYMS*) were significantly associated with specific immune cell infiltration. Among them, *ADA* was positively correlated with cytotoxic lymphocytes; *NT5E* was positively correlated with fibroblasts. Combined with existing studies, *ADA* not only participates in adenosine metabolism but also enhances T-cell activation and inflammatory factor release through binding to CD26 in the immune synapse, exerting a non-enzymatic immune co-stimulatory function.^39^ The study indicates that high expression of *ADA* may facilitate the activation of T cells and enhance anti-tumour immunity. On the contrary, *NT5E* (CD73) catalyses the generation of immunosuppressive adenosine from *AMP*, promoting the formation of an immunosuppressive microenvironment,^46,47^ which is consistent with the result that this gene is positively correlated with fibroblast infiltration. The results suggested that CD73 may promote the progression of OSCC by activating fibroblasts, promoting matrix remodelling and immunosuppression. *TYMS*, as a key enzyme in purine/pyrimidine metabolism, its excessive activation can enhance the DNA synthesis and proliferation of tumour cells, thereby changing the metabolic state of the tumour microenvironment and leading to the inhibition of immune cell function and tumour immune tolerance.^30^ In conclusion, this study systematically reveals the infiltration characteristics and potential functions of various immune cells in the OSCC tumour microenvironment, and indicates that metabolism-related genes such as *ADA*, *NT5E* and *TYMS* may affect the activity of immune cells and the tumour immune microenvironment by regulating adenosine metabolism and energy balance, thereby influencing the occurrence and development of OSCC. These findings provide a new theoretical basis and potential targets for in-depth exploration of the metabolic-immune interaction mechanism and the development of metabolic-targeted immunotherapy strategies for OSCC.

The drug analysis of hub genes showed that BAY-2402234, oxaliplatin, denileukin diftitox ontak, fluorouracil, and STREPTOZOCIN had lower IC50 values with increasing expression of *NT5E*. BAY-2402234 is a novel dihydroorotate dehydrogenase inhibitor and has recently been regarded as an inhibitor to effectively treat subtypes of acute myeloid leukaemia.^8^ However, its impact on OSCC treatment is limitedly studied. Oxaliplatin, a platinum-based chemotherapy drug mainly applied in the treatment of colorectal cancer (CRC) and other colon cancers,^35,37^ is proven to reinforce OSCC cell death by inducing the production of reactive oxygen species (ROS).^32^ Low-dose (5 μM) oxaliplatin may induce EMT and reinforce tumour metastasis in OSCC cells, while high-dose (40 μM) oxaliplatin exhibits inhibitory effects on tumours. Therefore, caution should be exercised when selecting oxaliplatin as a therapeutic agent for OSCC in terms of dosage.^41^ 5-FU is an effective inhibitor of *Clostridium difficile*, mainly used for the treatment of CRC29. Yang et al^60^ made an interesting discovery that extracellular vesicles derived from bitter melon reduced the resistance of 5-FU, which was originally effective for OSCC, allowing it to exert a greater effect. A study showed that the combination of docetaxel, cisplatin, and pembromycin with 5-FU chemotherapy is a safe and effective treatment for stage II-IV OSCC patients.^14^ The IC50 value of carboplatin increases with the elevation of the *ADA* level. Carboplatin is an organic platinum-based antitumour alkylating agent.^61^ A study on unresectable OSCC patients revealed that triple oral rhythm chemotherapy with paclitaxel and carboplatin is a novel adjuvant chemotherapy regimen that provides good survival rates for OSCC patients, with an OS of 63.5% at 15 months.^21^ Although this study predicted candidate drugs corresponding to NMRG-related characteristic genes in OSCC, some drugs have not been studied for their mechanisms or clinical trials in OSCC. Therefore, whether these drugs can be used for the treatment of OSCC requires further research on their functions and mechanisms in the future.

In conclusion, this study, for the first time, systematically analyses the relationship between OSCC and NMRGs. The hub genes *ADA*, *NT5E*, and *TYMS* that we discovered are correlated with the occurrence and development of OSCC, as well as immune cell infiltration and potential drug sensitivity. These results suggest that *ADA*, *NT5E*, and *TYMS* can serve as potential diagnostic markers and therapeutic targets for OSCC. The study provides a new reference for the early detection and individualised treatment of OSCC. This study has provided valuable findings; however, there are still some limitations. Firstly, this study mainly relied on bioinformatics analysis of public databases and lacked validation in an independent prospective clinical cohort as well as experimental verification of the hub genes through qPCR, immunohistochemistry, or functional experiments. Therefore, the clinical feasibility of the conclusions still needs further confirmation. Secondly, the innovation of this study mainly lies in systematically integrating NMRGs-related data, identifying OSCC-specific hub genes, and analysing their immunological relevance and potential drug targets, which is beyond the simple discovery of new genes. Finally, the drug sensitivity analysis results are derived from computational predictions in an *in vitro* database and have not been verified through *in vitro* experiments or clinical trials. Therefore, future evaluation of the practical application value of these hub genes in the diagnosis and treatment of OSCC needs to be further conducted by combining functional experiments and clinical data.

## REFERENCES

## Appendix

Appendix Tables A1 to A4 (https://www.quintessence-publishing.com/quintessenz/journals/articles/downloads/ohpd_2026_8002_wei_tables_s1_s4.xls)

**Fig A1:** ROC curve of the hub gene in the GSE30784 data set.

**Fig A2a to d:** Validation of the expression levels of key genes and exploration of their independent prognostic value. (a, b) Expression of key genes in normal and cancer samples of the GSE25099 data set (a) and GSE30784 data set (b). P > 0.05; * indicates P < 0.05; ** indicates P < 0.01; *** indicates P < 0.001; ns indicates no significant difference. (c, d) Forest plots of univariate (c) and multivariate (d) Cox regression analyses of key genes combined with clinical characteristics (age, gender, TNM stage, tumour stage).

**Fig A3a to d:** Screening of key lncRNAs and survival analysis. (a) Venn analysis of the intersection of differentially expressed lncRNAs in the TCGA data set and the lncRNAs predicted by the ENCORI database. (b) Univariate Cox regression analysis of the common lncRNAs obtained from the Venn analysis. (c) Kaplan–Meier survival analysis of lncRNA FOXD2.AS1. (d) Kaplan–Meier survival analysis of lncRNA AC156455.1.






